# Cyanine 5.5 Conjugated Nanobubbles as a Tumor Selective Contrast Agent for Dual Ultrasound-Fluorescence Imaging in a Mouse Model

**DOI:** 10.1371/journal.pone.0061224

**Published:** 2013-04-18

**Authors:** Liyi Mai, Anna Yao, Jing Li, Qiong Wei, Ming Yuchi, Xiaoling He, Mingyue Ding, Qibing Zhou

**Affiliations:** 1 Department of Nanomedicine & Biopharmaceuticals, National Engineering Research Center for Nanomedicine, Huazhong University of Science and Technology, Wuhan, Hubei, China; 2 Department of Biomedical Engineering, Huazhong University of Science and Technology, Wuhan, Hubei, China; 3 University Hospital, China University of Geoscience, Wuhan, Hubei, China; 4 Department of Medicinal Chemistry, Virginia Commonwealth University, Richmond, Virginia, United States of America; NIH, United States of America

## Abstract

Nanobubbles and microbubbles are non-invasive ultrasound imaging contrast agents that may potentially enhance diagnosis of tumors. However, to date, both nanobubbles and microbubbles display poor in vivo tumor-selectivity over non-targeted organs such as liver. We report here cyanine 5.5 conjugated nanobubbles (cy5.5-nanobubbles) of a biocompatible chitosan–vitamin C lipid system as a dual ultrasound-fluorescence contrast agent that achieved tumor-selective imaging in a mouse tumor model. Cy5.5-nanobubble suspension contained single bubble spheres and clusters of bubble spheres with the size ranging between 400–800 nm. In the in vivo mouse study, enhancement of ultrasound signals at tumor site was found to persist over 2 h while tumor-selective fluorescence emission was persistently observed over 24 h with intravenous injection of cy5.5-nanobubbles. In vitro cell study indicated that cy5.5-flurescence dye was able to accumulate in cancer cells due to the unique conjugated nanobubble structure. Further in vivo fluorescence study suggested that cy5.5-nanobubbles were mainly located at tumor site and in the bladder of mice. Subsequent analysis confirmed that accumulation of high fluorescence was present at the intact subcutaneous tumor site and in isolated tumor tissue but not in liver tissue post intravenous injection of cy5.5-nanobubbles. All these results led to the conclusion that cy5.5-nanobubbles with unique crosslinked chitosan–vitamin C lipid system have achieved tumor-selective imaging in vivo.

## Introduction

Nanobubbles and microbubbles as soft shell spheres containing inert gases such as perfluorocarbons or sulfur hexafluoride are contrast agents developed for non-invasive ultrasound imaging [1]–[3]. The enhanced ultrasound imaging signals have been attributed to the elastic compression and expansion of bubbles upon irradiation, resulting in modulation of reflected sound waves [1]–[3]. Nanobubbles and microbubbles have recently been investigated in the diagnosis and prognosis of tumors to reveal tumor vascular structures [4]–[6]. Compared to microbubbles, lipid nanobubbles with size below 500 nm have been reported to produce better ultrasound imaging enhancement [7]–[11] due to the enhanced permeation and retention (EPR) effects at tumor vascular leaks with pore size up to 780 nm [12]. However, to date, both nanobubbles and microbubbles display poor in vivo tumor-selectivity and have a short life of 30 min or less in vivo after being intravenously delivered [7]–[11]. For example, in addition to tumor imaging, phospholipid nanobubbles recently reported by Yin and coworkers produced extensive and strong ultrasound imaging enhancement in normal kidney and liver tissues in vivo, indicating poor tumor selectivity even with EPR effects [11]. One strategy to achieve the tumor selectivity is to use specific ligand-conjugated bubbles such as cyclic RGD peptides targeting tumor angiogenesis [13]–[15]. Nevertheless, the complex vascular structure and multiple growth markers of tumors at different stages pose a significant challenge to the effectiveness of this tumor-selective delivery strategy [16], [17]. Park and coworkers recently suggested that for any nano-drug delivery system, the tumor selective delivery over non-targeted organs must be first demonstrated in vivo, rather than on the assumption of the EPR effects by the size of nanoparticles [18]. The non-specific distribution of reported lipid nanobubbles may be attributed to the chemical composition of shell materials used, mostly neutral lipids and polyethylene glycol modified derivatives [7]–[11]. Thus, we hypothesized that using a negatively charged lipid as the major shell component such as ascorbyl palmitate could potentially change the in vivo distribution profile of nanobubbles.

Dual functional contrast agent has a potential to further enhance the diagnosis of tumor [19], [20], especially with fluorescence imaging that has been used in the application of image-guided resection of tumors to increase overall survival [21]–[23]. Fluorescence bubbles have been reported with quantum dots, chemical dyes using tumor-specific ligands such as folate or antibody [24]–[28]. However, most of them focused on the in vitro cell labeling while no effective in vivo tumor selectivity has been demonstrated. More recently, nanobubble lipid shells are found to have potential toxic effects on kidney and liver and interfere with immune responses [29], [30]. There is also safety concern of excess perfluorocarbon gas in the blood due to its low dissolving rate [3]. Thus, tumor-selective delivery is highly desirable to prevent the potential toxic issues of excess nanobubbles in circulation.

We report here a biocompatible chitosan-vitamin C lipid nanobubble system, cyanine 5.5-conjugated nanobubbles (cy5.5-nanobubbles) as a dual ultrasound–fluorescence contrast agent that achieved tumor-selective imaging in vivo. Fluorescence cy5.5 has maximum excitation and emission in the near infrared region at 675 and 690 nm, respectively and is commonly used in in vivo optical imaging due to low background interference. Cy5.5-nanobubbles were synthesized by sonication method with fully characterized physical properties. The enhancement of ultrasound and fluorescence signals at the tumor site was assessed in vivo post intravenous injection of cy5.5-nanobubbles. In vitro study of cellular uptake of cy5.5 was also investigated in cancer cells with nanobubbles. Finally, tumor selective imaging over non-targeted organ was assessed by both in vivo and tissue studies of the tumor mouse model post cy5.5-nanobubble injection.

## Materials and Methods

### Ethics Statement

The animal protocol was approved by the Animal Care and Use Committee of College of Life Science and Technology at Huazhong University of Science and Technology.

### Materials

All chemicals were purchased from Sigma-Aldrich (USA), J&K Scientific Ltd. (China) or Sinopharm Chemical Reagent Co., Ltd (China) unless otherwise specified. Murine liver cancer cells H22 were from Shanghai Institute of Life Science Cell Culture Center (China), and human liver cancer cell line Hep3B was obtained from ATCC (USA). Cells were maintained in RPMI-1640 or high glucose DMEM medium (Invitrogen, USA) supplemented with 10% heat-inactivated fetal bovine serum (FBS), 25 mM HEPES, 2 mM L-glutamine, 0.1 mM nonessential amino acids, 1.0 mM sodium pyruvate, 50 U/mL penicillin and 50 µg/mL streptomycin at 37°C and 5% CO_2_.

### Synthesis of cy5.5-nanobubbles

Ultrasound generation of nanobubbles was carried out on a JY99-II DN ultrasound homogenizer with a Ø20 mm horn (Ningbo Scientz Biotechnology Co. Ltd, China). To a suspension of hydroxyethyl starch (200/0.5, 1.20 g, Wuhan HUST Life Science & Technology Co. Ltd., China), ascorbyl palmitate (220 mg), 1,2-hexadecandiol (6 mg) in sterile phosphate buffer saline solution (PBS, pH 7.4, 58 mL) were added solutions of tween-60 in PBS (3% w/w, 2 mL) and then 1 N NaOH (300 µL). The resulting mixture was cooled down to 10°C in an ice bath and degassed for 10 min with 0.2 µM filtered perfluoropropane gas (C_3_F_8_, 99.7%, Institute of Physical and Chemical Engineering in Nuclear Industry & Huahei Technology Development Co., Ltd., China). For the sonication process, the tip of ultrasound horn was lowered approximately 2 mm below the surface of the solution to ensure effective mixing of particles and gas. The suspension was sonicated under C_3_F_8_ gas at 540 W for 80 cycles with working and resting time of 5 and 10 s per cycle, respectively. The temperature of the solution was maintained below 15°C with an ice bath throughout the entire sonication process. A chitosan solution (low molecular weight, 0.5% w/w in 0.5% acetic acid aqueous solution, 150 µL) was then slowly added to the mixture under sonication at 36 W. The suspension was sonicated again at 36 W for 40 cycles with working and resting time of 5 and 10 s per cycle, respectively. The resulting milky solution (∼50 mL) was centrifuged at 250×g for 45 min at 4°C in a 50 mL conical tube. The middle section of obtained supernatant was slowly drained to vials and sealed under C_3_F_8_ (approximately 30 mL). Cy5.5-nanobubble suspension (5 mL each) were obtained by adding cy5.5 N-hydroxysuccinimide ester in DMSO (20 mg/mL, 0.6 µL, GE Health, USA) and then a crosslinker bis(succinimidyl) penta(ethylene glycol) (BS(PEG)_5_, Thermo Scientific, USA) in DMSO (1.5 mg/mL, 10 µL) after 12 h. The pH of the final Cy5.5-nanobubble suspension was measured as 7.2. To determine the percentage of conjugated cy5.5 on nanobubbles, cy5.5-nanobubble suspension (1 mL each×3) was centrifuged at 10,000 g×10 min, the UV absorbance of free cy5.5 in the supernatant was measured at 688 nm as compared to a standard curve. The percentage of conjugation was calculated as 41% based on the molar ratio of free cy5.5 detected in supernatant over the total activated ester of cy5.5 added.

As a control solution for cy5.5-nanobubbles, cy5.5 free acid plus nanobubble mixture (cy5.5+nanobubbles) was obtained by first synthesis of nanobubble suspension (5 mL each) without the addition of cy5.5 *N*-hydroxysuccinimide ester as described above. Cy5.5 free acid (20 mg/mL in DMSO, 0.6 µL, GE Health, USA) was then added in 5 mL nanobubble suspension. Another control solution for cy5.5-nanobubbles was the cy5.5 conjugated chitosan solution (cy5.5-chitosan) as the shell material without nanobubble structure. Cy5.5-chitosan solution was generated by adding low molecular chitosan solution (0.5% w/w in 0.5% acetic acid aqueous solution, 12.5 µL) in PBS solution (5 mL) containing 2% hydroxyethyl starch (200/0.5) followed by addition of cy5.5 N-hydroxysuccinimide ester (20 mg/mL in DMSO, 0.6 µL) and then BS(PEG)_5_ crosslinker (1.5 mg/mL in DMSO, 10 µL) as described above.

### Characterization of cy5.5-nanobubbles

Microscopic imaging analysis was carried out on a Nikon Eclipse 80i microscope equipped with a Plan Apochromat VC 100× oil objective lens (Nikon Instrument, Japan). Cy5.5-nanobubble suspension (50 µL) was diluted with PBS solution (100 µL) and loaded onto a hemocytometer with cover slides. The field of view was first adjusted to the center of hemocytometer that has gridded squares of a size of 50 µm under a 40× objective lens. Images and videos of nanobubbles were then recorded under the 100× oil objective lens with Nikon NIS-Elements BR imaging capturing software. The scale of the field of view was calibrated with the average size of gridded squares. Microscopic cy5.5 fluorescence image of nanobubbles was recorded similarly with IX71 Inverted Microscope (Olympus Corporation, Japan) equipped with a digital camera.

The hydrodynamic size measurement of nanobubbles was carried out with a Nano-ZS90 particle analyzer (Malvern, United Kingdom). Nanobubble suspension (200 µL) was mixed in PBS solution (1.25 mL), and the hydrodynamic size and zeta potential were recorded after equilibration at 37°C for 5 min. The excitation and emission spectra of cy5.5-nanobubble suspension were obtained in PBS solution (200 µL in 2 mL) using Hitachi F-4500 fluorescence spectrometer (Tokyo, Japan) at room temperature (Figure S1-a).

### Ultrasound Imaging Property of cy5.5-nanobubbles

The setup for analysis of ultrasound reflection by cy5.5-nanobubbles included a latex glove fingertip containing 10 mL PBS solution in a water bath with ultrasound transducer on one side. Ultrasound images were recorded on a LOGIQ 7 Ultrasound System (GE Healthcare, USA) in B flow mode with a thyroid transducer. Solutions in the latex finger and water bath were first verified to be free of bubbles by ultrasound imaging. Cy5.5-nanobubble suspension (150 µL) was then injected into the PBS solution inside the latex finger at the bottom, and ultrasound images were captured at room temperature.

### In vitro Cellular Fluorescence Study

Hep3B cells (20,000 per well) were seeded in the growth media (400 µL) on a 48-well plate overnight. A solution of cy5.5+nanobubbles, cy5.5-chitosan or cy5.5-nanobubbles (10 µL each) was then added to each well and mixed gently with cell media. After incubation for 3 h at 37°C, cell media were removed and cells were washed once with PBS. Images of each treatment under phase light and with cy5.5 fluorescence emission were captured with IX71 Inverted Microscope equipped with a digital camera.

### Mouse Tumor Model

SFP nude female BALB/c mice (approximately 20 g) were obtained from Hunan Slake Jingda Experimental Animal Co. Ltd., China. Murine liver cancer cells H22 were grown in the BALB/c mice intraperitoneally. Mice were euthanized after 6 days and H22 cells were harvested with PBS solution. H22 cells were washed once with sterile PBS and were injected subcutaneously (3×10^6^ cells per mouse) at the lower back of nude BALB/c mice [31]. Typically, tumors reached an average size of 0.8×0.6 cm after 7 days post injection, and mice were used in the following imaging analyses.

### In vivo Ultrasound and Fluorescence Imaging

For ultrasound imaging of subcutaneous tumors, mice were restrained on a flat platform without anesthetics. Ultrasound transducer was first positioned gently on the top of tumors parallel to the direction of spinal cord and then at an orthogonal angle. Ultrasound images were recorded on a LOGIQ 7 Ultrasound System with a thyroid transducer at a frequency of 12 MHz prior and post a single intravenous injection of cy5.5-nanobubble suspension (4 µL per gram of body weight) through the tail vein. Mice were then released and allowed to rest in the intervals between time points of 10, 30, 60, 120 and 240 min post injection. A total of 4 mice were used for in the ultrasound study.

For the in vivo fluorescence imaging analysis, the optimal excitation wavelength for cy5.5 emission in nude mice was first assessed to be 640 nm on a naïve mouse with subcutaneous injection of 100 µL cy5.5-nanobubble suspension. During the fluorescence imaging, mice were under gas anesthesia with oxygen and isoflurane (Hebei Jiupai Pharmaceuticals, China). Images were obtained prior and post a single intravenous injection of cy5.5+nanobubble, cy5.5-chitosan or cy5.5-nanobubble suspension (4 µL per gram of body weight) through the tail vein (3 mice per group). Images were captured with IVIS® Lumina XR Imaging System (Caliper Life Sciences, USA) using a set of imaging sequence including photograph and fluorescence modes with excitation wavelengths at 640 nm and cy5.5 emission filter. Fluorescent images in radiation efficiency were processed with Living Image® software according to manufacturer’s recommendation. Briefly, cy5.5 fluorescence emission image of mice was obtained with spectral unmixing method to remove background emission that was obtained at excitation at 535, 570, or 605 nm. Folds of fluorescence increase at tumor sites over time were calculated by the ratio of total radiation efficiency over a uniformly defined area pre- and post-injection with Living Image® software.

For fluorescence imaging of isolated tumors and tissues, mice were euthanized after 24 h post intravenous injection of cy5.5-nanobubble or PBS solution. The skin containing tumor was exposed for imaging. The tumor and liver tissues were then isolated and fluorescence images were obtained and processed similarly as described above.

## Results and Discussion

### Synthesis of cy5.5-nanobubbles

Ascorbyl palmitate was chosen as a major component for the new formulation of nanobubbles due to the facts that it is a highly biocompatible vitamin C lipid and has a negative charge at physiological pH that can stabilize bubbles via ionic interactions. The generation of nanobubbles was initially carried out by sonication of a solution of 0.37% ascorbyl palmitate in phosphate buffer saline solution (PBS) under perfluoropropane gas with only 0.1% tween-60 that was able to help the formation and stabilization of nanobubbles. Nanoscale bubbles were found only with two rounds of sonification at power of 540 W and then 36 W as indicated by dynamic light scattering analysis (DLS). However, the resulting nanobubble suspension was not stable over a period of 24 h at 4°C with precipitations.

**Figure 1:**
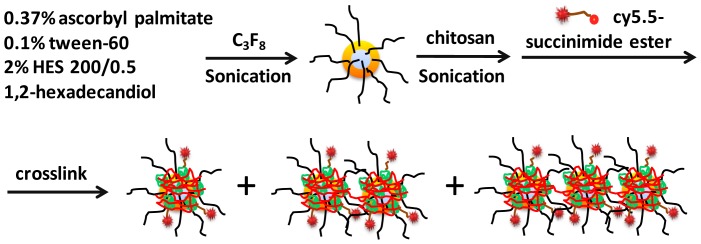
Stepwise synthesis of cy5.5-nanobubble suspension.

Stabilization of nanobubbles was achieved by first adding a dihydroxylated lipid that markedly reduced precipitations in nanobubble suspension possibly via enhanced hydrophobic interaction in the lipid layer. Secondly, chitosan is a polymer of glucosamines containing amino groups with average p*K_a_* of 6.5 and are positively charged under neutral pH. Addition of chitosan at pH 7 stabilized nanobubbles via ionic interaction with the negative charged ascorbate surface of nanobubbles. In addition, chitosan was also crosslinked to enhance such stabilization through amide bond formation. Finally, addition of water soluble hydroxylethyl starch (200/0.5), a major constituent of blood substitutes used in clinic, provided a highly branched polymeric structure to reduce potential cluster formation of nanobubbles via hydrogen bonds with chitosan shell. Thus, optimized synthesis of stable cy5.5-nanobubbles was achieved and summarized in Figure 1. Briefly, nanobubbles were generated with 1,2-hexadecandiol in the presence of hydroxylethyl starch in PBS solution. Chitosan was added prior to the second round sonication while crosslinker BS(PEG)_5_ was added to nanobubble suspension at 0.25 equivalent to the glucosamine unit of chitosan. Incorporation of fluorescence cyanine 5.5 (cy5.5) on nanobubbles was carried out before the crosslink process. The resulting cy5.5-nanobubble suspension was found to be stable over 6 weeks at 4°C.

### Characterization of cy5.5-nanobubbles

**Figure 2:**
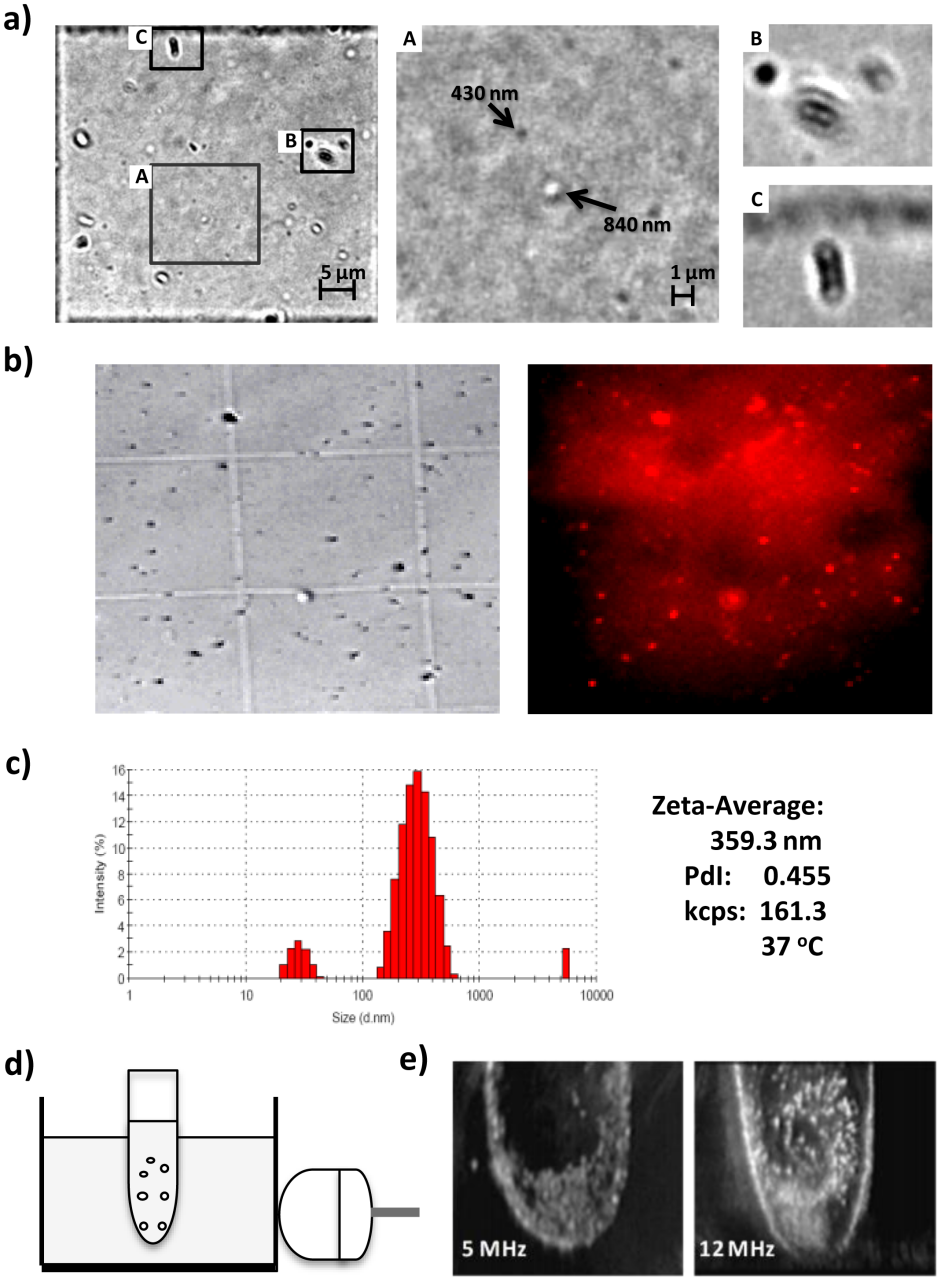
Physical properties of cy5.5-nanobubbles. a) microscopic images of cy5.5-nanobubbles in a 50 µm grid square of a hemocytometer under 100× objective lens with A, B and C as expanded views of selected areas in the left panel; b) microscopic images of cy5.5-nanobubbles on a hemocytometer under phase light (left panel) or with cy5.5 emission filter (right panel); c) hydrodynamic sizes of cy5.5-nanobubbles as obtained by dynamic light scatting measurement in PBS solution; d) schematic illustration of the setup for analysis of ultrasound imaging property of nanobubbles; e) ultrasound images of nanobubbles at 5 and 12 MHz.

Sizes of nanobubbles were first confirmed by microscopic analysis with both static and video imaging. Static microscopic imaging analysis has been applied successfully to determine size, structure and density of microbubbles but not for sub-microscale bubbles [32]. We found that with the use of an oil-immersed 100× objective lens, the resolution of microscopic imaging analysis could be extended to 0.07 µm/pixel that was sufficient for analysis of particle sizes above 300 nm. Microscopic images of nanobubbles in a 50 µm grid square of a hemocytometer under the 100× objective lens are shown in Figure 2a. Diameters of nanobubbles were estimated from the average number of pixels along two orthogonal axes. Three types of particles were observed including grey solid spheres, white hollow circles and clusters of beads. Expansion of selected areas in the left panel (Figure 2a-A) indicated that grey spheres and white hollow circles were particles around 400 and 800 nm, respectively while clusters were typically groups of hallow circles (Figure 2a-B and C). Microscopic analysis of recorded video of nanobubbles under the 100× objective lens (Video S1) revealed that all particles were in constant vibrational motion in and out of the field of view, which made the estimation of the particle density of the solution infeasible. More importantly, a single bubble could be observed either as a grey solid sphere when it moved away from the focus of the field or as a white hollow circle when it moved toward focus of the field. Thus, the hollow white circle may be the result of self-magnification by light scattering through the bubble structure. In addition, cluster of beads were indeed linked spheres that vibrated together. Therefore, microscopic imaging analysis at high magnification confirmed that synthesized nanobubbles were spherical bubbles and clusters of these bubbles with the size ranging between 400–800 nm. The cluster formation of bubbles was found as the result of addition of chitosan solution following by the crosslink agent, possibly due to the covalent linkage of multiple single bubbles. The incorporation of cy5.5 on nanobubbles was then verified with microscopic imaging method. The image obtained under phase light matched closely with the red fluorescence color located on the bubbles in the image captured with cy5.5 emission filter (Figure 2b). In contrast, the control of cy5.5+nanobubbles only resulted in a blur red fluorescent background. This result suggested that cy5.5 dye was covalently attached to the surface of nanobubbles.

Hydrodynamic sizes of cy5.5-nanobubbles were determined with dynamic light scattering analysis as a complementary method to microscopic analysis (Figure 2c). The zeta average size was 359.3 nm in PBS solution at 37°C in the range between 200–800 nm with a polydispersity distribution of particle sizes (PDI) at 0.46. Nanobubbles were found to have a negatively charged surface with zeta potential of −19.17 mV. The formation of nanobubble clusters may be responsible for the relative high polydispersity index of nanobubble suspension as a polydispersed system. Further separation of nanobubble suspension with centrifugation did not result in significant removal of these nanobubble clusters. Therefore, the above synthesized nanobubble suspension was used in the following physical and biological analyses. In addition, the count rate was found to remain approximately at 161×10^3^ particles per second during the DLS measurement with a 6.25-fold diluted sample, and thus was used as an index to represent the nanobubble density of the synthesized suspension as 1.01×10^6^ bubbles per second. Additional characterization of nanobubbles with transmission electron microscopic analysis was not successful because synthesized cy5.5-nanobubbles were ruptured under experimental condition used. As a comparison, the cy5.5 free acid plus nanobubbles control has a zeta average hydrodynamic size of 382.4 nm with a PDI of 0.50 (Figure S1-b), indicating that covalent conjugation of cy5.5 on the nanobubbles did not significantly increase the sizes of nanobubbles.

The enhanced reflection of ultrasound by cy5.5-nanobubbles in ultrasound imaging was first assessed in a latex finger as shown schematically in Figure 2d. Ultrasound imaging upon injection of nanobubble suspension was evaluated at a frequency of 5 or 12 MHz with the same setting of instrumental parameters (Figure 2e). Cy5.5-anobubbles produced a strong ultrasound signal as bright white spots in both images as compared to the low signal dark background. On the other hand, the enhanced ultrasound signal at a frequency of 12 MHz was much brighter than that at 5 MHz under the same condition. The enhancement of ultrasound signal by bubbles correlates to their harmonic frequency, which generally follows Rayleigh-Plesset-like equation and depends on the shell material, inner and outside diameters and interaction with the media [33]–[35]. Thus, the enhanced contrast by cy5.5-nanobubbles at a high frequency may reflect a unique elasticity due to the crosslinked chitosan-lipid ascorbate shell structure. This unique elasticity of cy5.5-nanbubbles may also explain why the ultrasound image with cy5.5-nanbubbles in the B mode had a slightly better contrast than that in the inverted phase harmonic mode, the latter of which is commonly used for micrcobubbles. Therefore, the synthesized nanobubbles were able to enhance ultrasound signals in ultrasound imaging. Based on these results, the following in vivo ultrasound imaging studies were carried in the B-flow mode at the frequency of 12 MHz.

### Tumor Selective Imaging with cy5.5-nanobubbles

**Figure 3:**
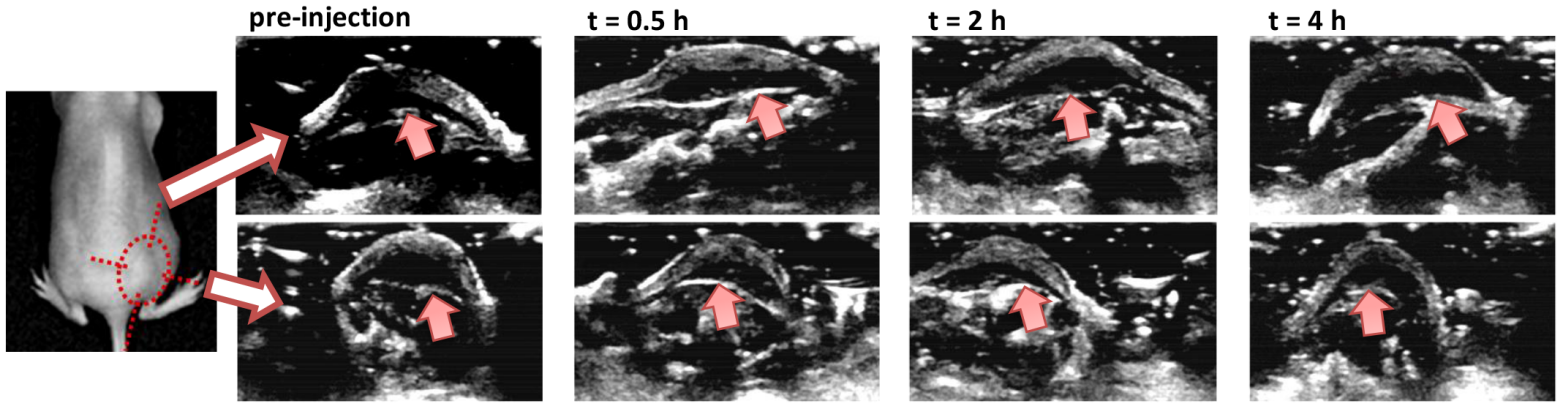
In vivo ultrasound images of subcutaneous tumors with injection of cy5.5-nanobubble suspension. Ultrasound images were obtained with LOGIQ7 system with a thyroid transducer at 12 MHz. The location of subcutaneous tumor was marked with red circle in the left panel. Top and bottom panels are images of the same tumor at orthogonal angles. The basal periphery of tumor was indicated with arrows in images. Images are representative of 4 mice investigated.

Cy5.5-nanobubbles as a dual ultrasound-fluorescence contrast agent were then assessed in a mouse tumor model. Subcutaneous tumor was established in nude mice at lower back with an average size of 0.8×0.6 cm. The ultrasound images of tumor were obtained at two orthogonal angles prior injection of cy5.5-nanobubble suspension as indicated in Figure 3. Under ultrasound, the subcutaneous tumor appeared as an irregular hill shape with the skin as the top bright contour and tumor as a dark hypoechoic area. In general, an irregular hypoechoic dark area with fully enclosed periphery in two orthogonal ultrasound images is indicative of a malignant tumor in B mode [36], [37]. However, the basal periphery of tumor was poorly defined in the top panel of ultrasound image as compared that in the bottom panel (indicated with arrows in Figure 3), and thus requiring additional tests to confirm the presence of a malignant tumor.

Following an intravenous injection of cy5.5-nanobubble suspension in mice, ultrasound images of tumor were recorded over a period of 4 hours (Figure 3). While the tumor remained as a hyposonically dark area, the basal periphery of tumor became highly visible with strong ultrasound signals as a white line at both orthogonal angles at 30 min post injection (indicated with arrows in Figure 3). These results led to a consistent indication of the presence of a malignant tumor, suggesting that nanobubbles were able to enhance the diagnosis of subcutaneous tumor as a contrast agent. In addition, the enhancement of tumor basal periphery persisted strongly for 2 hours and then gradually decreased within next 2 hours. At 4 h post intravenous injection, the ultrasound images of tumor were found to be similar to those of prior injection (Figure 3). The loss of the ultrasound enhancement at 4 h suggested the absence of cy5.5-nanobubbles at the periphery of tumor site, possibly due to the rupture of nanobubbles in vivo as that of reported nanobubbles [7]–[11]. This result, on the other hand, confirmed that the increased contrast of tumor base periphery at early time points was a result of accumulation of cy5.5-nanobubbles at tumor site over a time of 4 h, much longer than that of reported lipid nanobubbles [7]–[11]. The penetration of cy5.5-nanobubbles into tumor stroma was not observed in the ultrasound imaging, which may be due to the relative large sizes of cy5.5-nanobubbles and possibly that the available ultrasound imaging mode was not optimized for cy5.5-nanobubbles. More importantly, in contrast to reported lipid nanobubbles, no significant enhancement by cy5.5-nanobubbles in the normal rat liver tissue was observed in vivo in ultrasound imaging (Figure S1-c), suggesting that cy5.5-nanobubbles was able to selectively accumulate in the periphery of tumor site over non-targeted organs such as liver. During and post the imaging process, no abnormal mouse behavior or mortality was observed after the injection of nanobubble suspension, suggesting the safety of cy5.5-nanobubbles via intravenous delivery.

**Figure 4:**
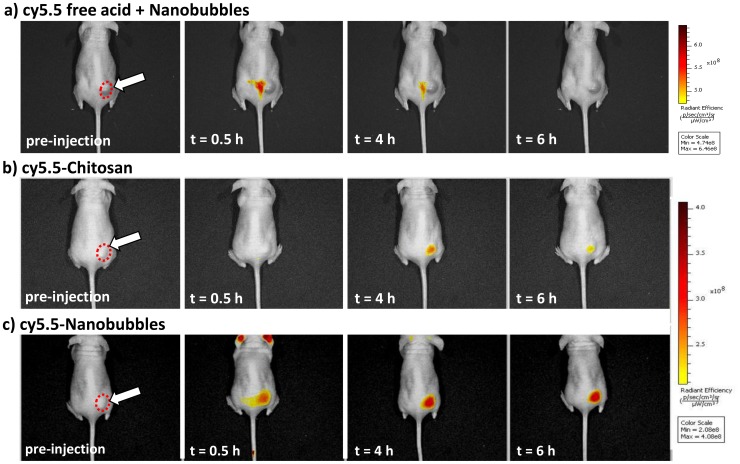
In vivo fluorescence images of mouse tumors model with cy5.5 solutions. Mice with average tumor sizes of 0.8×0.6 cm (marked with red circles with arrows in left panels) were injected intravenously with: a) cy5.5 free acid+nanobubbles; b) cy5.5-choitosan solution as a control of shell materials without nanobubble structure; and c) cy5.5-nanobubble suspension. For panels b and c, cy5.5 fluorescence emission is shown under the same scale of radiation efficiency for comparison of intensity at the tumor sites. Fluorescence images are shown as overlaid fluorescence emission profiles on photographic images.

The in vivo fluorescence images of the mouse prior and post intravenous injection of controls and cy5.5-nanobubbles are shown in Figure 4. Intravenous injection of free cy5.5+nanobubbles led to a general fluorescence signal at the back of the mouse after 30 min, which gradually disappeared over time post injection (Figure 4a). No significant fluorescence was detected at tumor site with cy5.5+nanobubble control. Additional fluorescence imaging analysis revealed that there was a high fluorescence signal in the mouse bladder at 2 h post injection of free cy5.5+nanobubbles, which was significantly decreased after 24 h (Figure S1-d). Our results suggested that free cy5.5 was likely removed by renal filtration from the blood circulation and excreted via mouse bladder. Injection of the cy5.5-chitosan control of shell material without nanobubble structures resulted in a weak cy5.5 emission at the tumor site (Figure 4b). The fluorescence intensity then decreased significantly at 6 h. In stark contrast, accumulation of red fluorescent emission was observed at the tumor site as early as 30 min post injection of cy5.5-nanobubbles and reached a maximum at 4 h. The increase of the fluorescence at tumor site concurred with the decrease of fluorescence emission of mouse ears where blood vessels are close to surface, suggesting accumulation of cy5.5-nanobubbles at tumor site and a decreased level in bloodstream. These results indicated that tumor selective cy5.5 fluorescence imaging could be only effectively achieved with covalently conjugated cy5.5-nanobubbles not with the mixture of free cy5.5+nanobubbles, and also that the nanobubble structure was critical for the tumor selective imaging.

**Figure 5:**
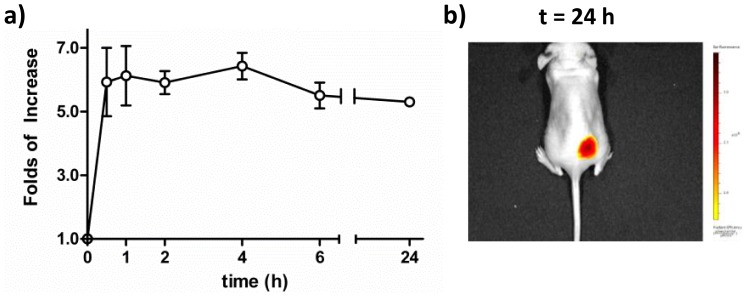
Persistent tumor-selective imaging with cy5.5-nanobubbles. a) Folds of in vivo fluorescence intensity enhancement at tumor site over 24 h; and b) fluorescence image of the mouse tumor site at 24 h post injection.

The accumulated cy5.5 fluorescence at tumor site with cy5.5-nanobubbles was consistent with the tumor selective ultrasound imaging in Figure 3. Interestingly, the highest intensity of cy5.5 fluorescence concurred with the disappearance of enhancement in the ultrasound imaging at 4 h post intravenous injection, suggesting that cy5.5-nanobubble shell material remained at the tumor site even though cy5.5-nanobubbles were ruptured. Thus, cy5.5-nanobubbles were an effective dual contrast agent for selective imaging of tumor in vivo. The fluorescence accumulation profile of the tumor site over a period of 24 h in vivo was summarized in Figure 5a. The fluorescence intensity of the tumor site reached approximately 6 folds of that prior injection in 4 hours and remained persistently high even after 24 h (Figure 5b).

**Figure 6:**
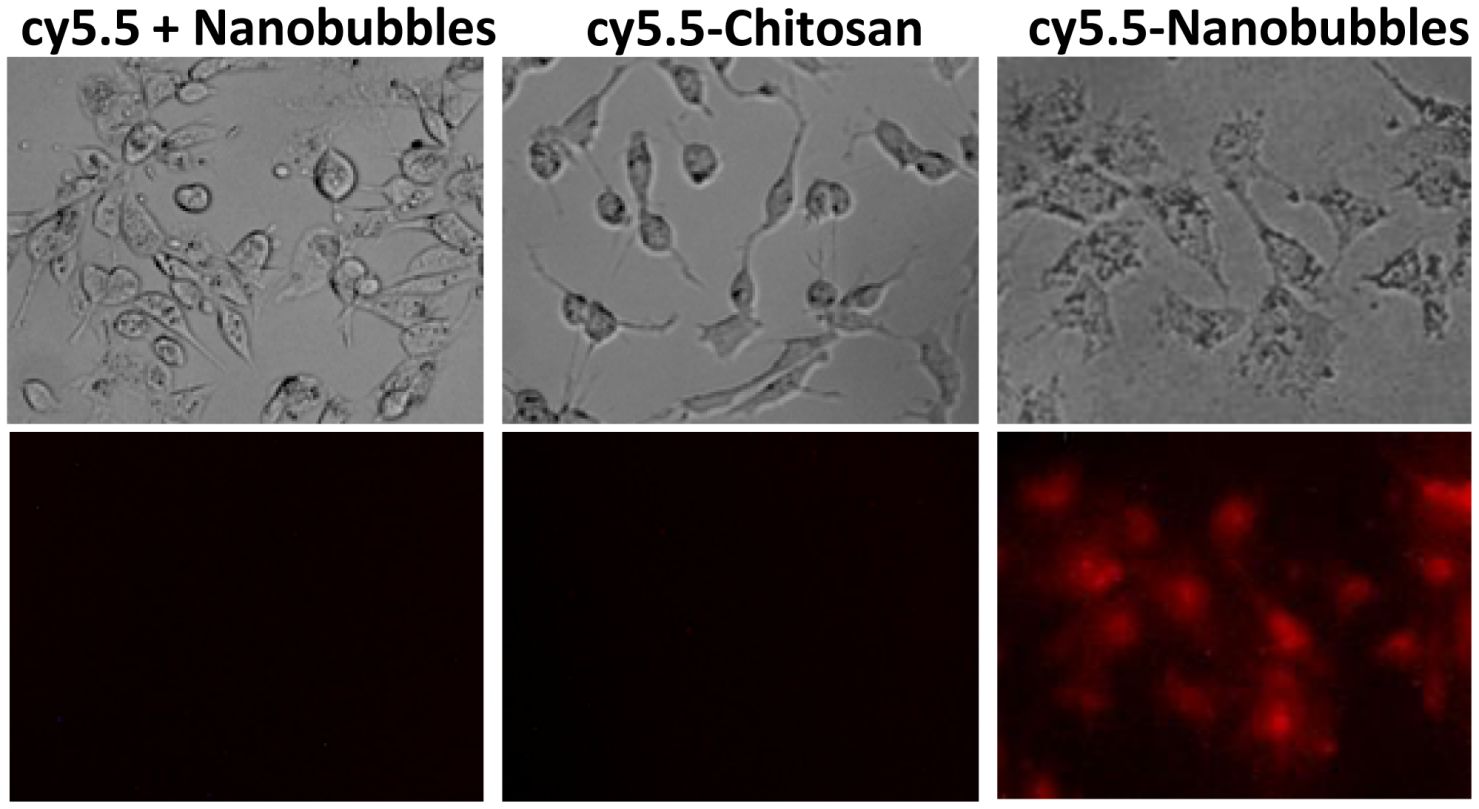
In vitro cellular uptake study of cy5.5-nanobubbles. Images of cellular accumulation of cy5.5 fluorescence in liver cancer Hep3B cells upon treatment of a) cy5.5 free acid+nanobubbles; b) cy5.5-chitosan solution; or c) cy5.5-nanobubbles for 3 h at 37°C. Top and bottom panels are images obtained under phase light and with cy5.5 emission filter, respectively.

In vitro study of cy5.5 cellular uptake was then investigated whether cy5.5-nanobubbles were able to accumulate selectively in tumor cells. Cell images under phase light and with cy5.5 emission filter are shown in Figure 6 after liver cancer Hep3B cells treated with cy5.5-nanobubble suspension or controls for 3 h. A low level of cy5.5 fluorescence emission signal was observed inside cells when treated with cy5.5-nanobubble suspension. In contrast, neither cy5.5+nanobubble nor cy5.5-chitosan control produced any red fluorescence inside cells. This result indicated that cy5.5-nanobubbles were able to enter in cells, which might explain why the red fluorescence accumulated selectively at tumor site in vivo. In addition, this cellular uptake result also suggested that cy5.5-chitosan as a shell material without nanobubble structures might not be adsorbed to tumor tissue in vivo. Taken together, these results suggested that only the cy5.5 conjugated crosslinked chitosan-lipid vitamin C nanobubbles could achieve tumor-selective imaging in vivo in contrast to other lipid or phospholipid nanobubbles [7]–[11].

**Figure 7:**
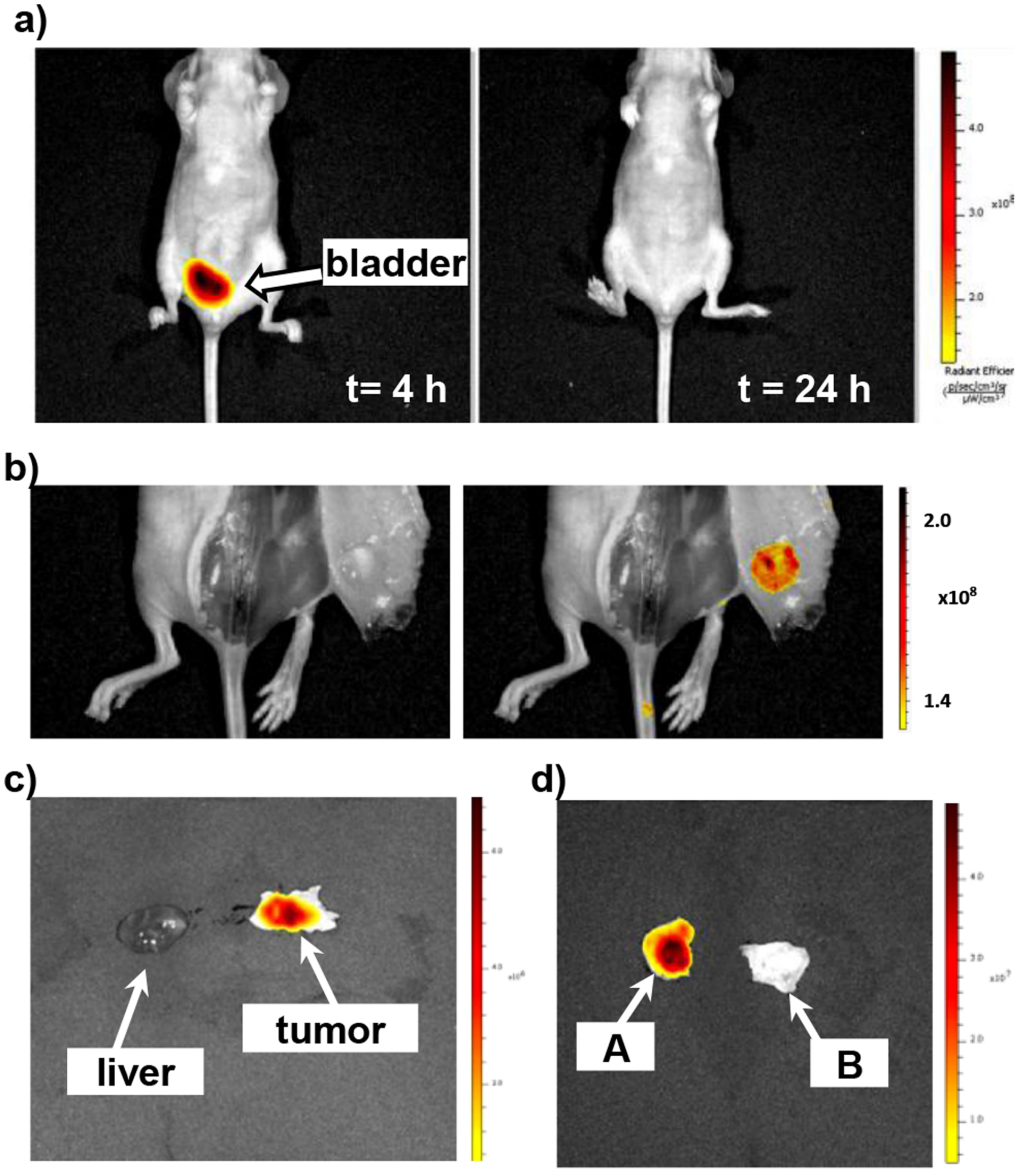
Selective accumulation of cy5.5-nanobubbles at mouse tumor site. a) In vivo fluorescence images of the front side of mouse at 4 and 24 h post injection revealing that bladder is the alternative location of cy5.5-nanobubbles (tumor is located at the backside); b) photograph and the cy5.5 fluorescence image of an intact subcutaneous tumor at 24 h post intravenous injection; c) comparison of cy5.5 fluorescence in isolated liver and tumor tissues of the same mouse at 24 h post intravenous injection; d) comparison of cy5.5 fluorescence in isolated tumor tissues with cy5.5-nanobubbles (A) or plain PBS injection (B) at 24 h post intravenous injection. Fluorescence images are shown as overlaid fluorescence emission profiles on photographic images.

The tumor-selective targeting by cy5.5-nanobubbles was then verified with both in vivo and collected tissue studies. Alternative location of cy5.5-nanobubbles was observed in the bladder from the front side of mouse at 4 h post injection (Figure 7, tumor is located at the backside) while no significant fluorescent emission was observed at the location of liver. The fluorescence intensity at bladder decreased significantly after 24 h. The fluorescence signal in the mouse bladder with cy5.5-nanobubbles might be due to the released cy5.5 in the nanobubble suspension that was removed by kidney as that of free cy5.5 control. The ratio of the fluorescence emission in tumor versus bladder at 4 h was estimated approximately as 1∶1.6 based on the area integration, indicating a quite efficient selectivity of tumor site in vivo. In addition, high intensity of cy5.5 fluorescence emission was found at the intact subcutaneous tumor underneath the skin at 24 h post intravenous injection of cy5.5-nanobubbles as compared to the photography of the tumor site (Figure 7b, left versus right panel). This result confirmed that in vivo fluorescence images at tumor site at early time points were indeed due to the presence of cy5.5-nanobubbles. Finally, there was dramatic higher emission intensity of cy5.5 fluorescence of isolated tumor tissues than that of liver tissues of the same mouse, confirming that liver was not a major accumulation organ for cy5.5-nanobubbles (Figure 7c). Moreover, the collected tumor tissue with cy5.5-nanobubble injection showed a marked 28-fold increase of total fluorescence intensity versus that with PBS injection (Figure 7d). All these results confirmed that cy5.5-nanobubbles were able to achieve tumor-selective targeting in vivo.

In conclusion, we present in this report cy5.5-nanobubbles as a dual ultrasound-fluorescence contrast agent for selective tumor imaging in vivo. Based on the results from the ultrasound and fluorescence imaging analyses, it is suggested that the tumor selectivity by cy5.5-nanobubbles could be attributed to the unique crosslinked chitosan-ascorbyl palmitate structure with a negatively charged surface. As shown by ultrasound imaging analysis (Figure 3), cy5.5-nanobubbles accumulated at the tumor site through the blood circulation in the initial 2 h post intravenous injection, and then degraded over next 2 h. Meanwhile, the fluorescence signal of cy5.5 conjugated shell materials could remain at tumor site over 24 h (Figures 4c, 5 and 7b–d). In contrast, free cy5.5 was quickly removed by renal filtration followed by excretion via bladder (Figures 4a and S1-d). The in-depth mechanisms of selective accumulation of cy5.5-nanobubbles at tumor tissue are currently under investigation. Future direction will be the investigation on whether the tumor selective imaging can be further enhanced when a tumor-targeting ligand or antibody is incorporated on the synthesized cy5.5-nanobubbles.

## Supporting Information

Figure S1 a) Fluorescence excitation and emission spectra of cy5.5-nanobubble suspension; b) hydrodynamic sizes of cy5.5 free acid+nanobubble suspension as obtained by dynamic light scatting measurement in PBS solution at 37°C; c) in vivo ultrasound images of the normal liver of a Sprague-Dawley rat pre- and post-iv injection of 200 µL of cy5.5-nanobubble suspension. Ultrasound images were obtained with LOGIQ7 system with a thyroid transducer at 12 MHz. The image post injection was obtained at 2 min and no significant enhancement of ultrasound signal was found afterwards; d) in vivo fluorescence images of the front side of mouse at 2 and 24 h post injection of free cy5.5+nanobubbles, confirming that cy5.5 was removed through renal filtration.
(TIF)

Video S1 A video clip of microscopic analysis of cy5.5-nanobubbles on a hemocytometer under a Plan Apochromat VC 100× oil objective lens with a Nikon Eclipse 80i microscope.
(WMV)
