# Supplementary figures and images for: Cyanine 5.5 Conjugated Nanobubbles as a Tumor Selective Contrast Agent for Dual Ultrasound-Fluorescence Imaging in a Mouse Model
Source: PLoS One. 2013 Apr 18;8(4):e61224. doi: 10.1371/journal.pone.0061224 (PMC3630137; doi:10.1371/journal.pone.0061224)

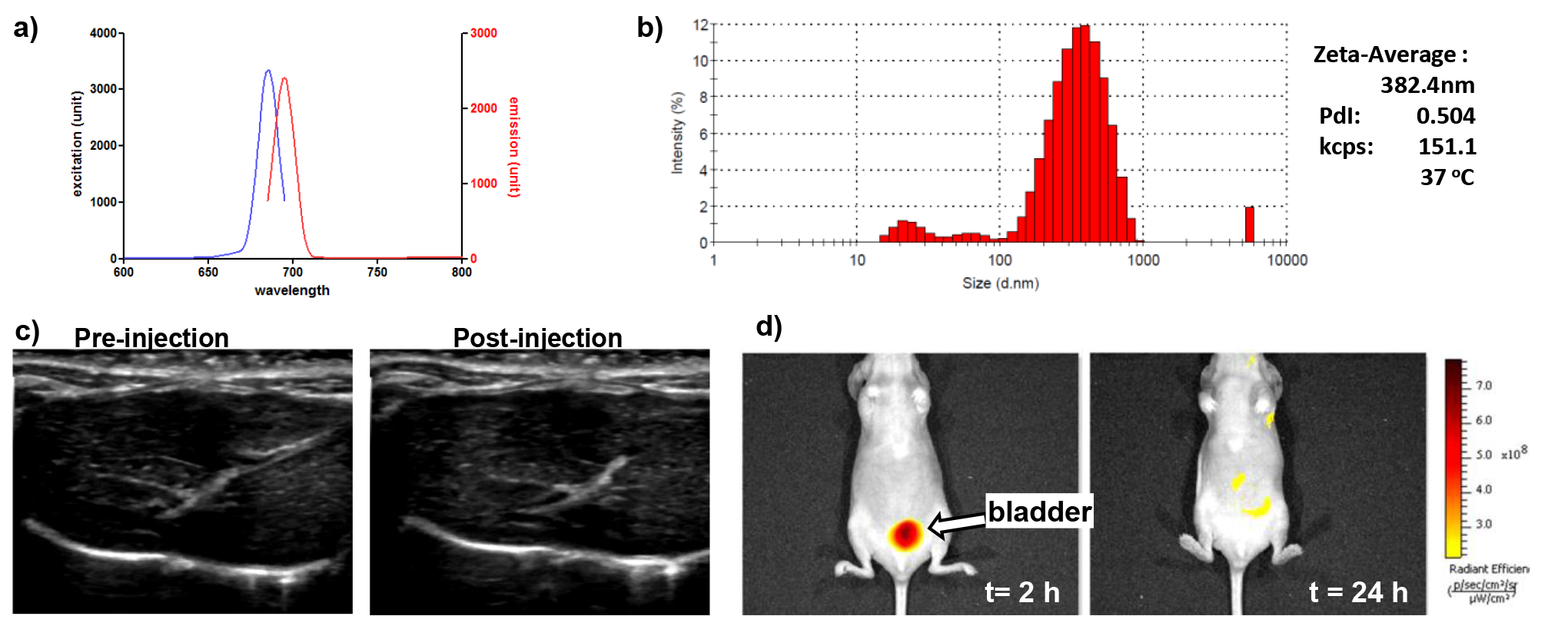

Supplement: Figure S1 — a) Fluorescence excitation and emission spectra of cy5.5-nanobubble suspension; b) hydrodynamic sizes of cy5.5 free acid+nanobubble suspension as obtained by dynamic light scatting measurement in PBS solution at 37°C; c) in vivo ultrasound images of the normal liver of a Sprague-Dawley rat pre- and post-iv injection of 200 µL of cy5.5-nanobubble suspension. Ultrasound images were obtained with LOGIQ7 system with a thyroid transducer at 12 MHz. The image post injection was obtained at 2 min and no significant enhancement of ultrasound signal was found afterwards; d) in vivo fluorescence images of the front side of mouse at 2 and 24 h post injection of free cy5.5+nanobubbles, confirming that cy5.5 was removed through renal filtration. (TIF) [file pone.0061224.s001.tif]
